# Fluoroscopy-guided direct anterior approach total hip arthroplasty provides more accurate component positions in the supine position than in the lateral position

**DOI:** 10.1186/s12891-023-07014-4

**Published:** 2023-11-14

**Authors:** Penglei Chen, Wangxin Liu, Cong Wu, Pengfei Ruan, Jicheng Zeng, Weifeng Ji

**Affiliations:** 1 The First Clinical Medical College, Zhejiang Chinese Medical University, 310006 Hangzhou, China; 2 The First Affiliated Hospital of Zhejiang Chinese Medical University, No9,9th Street, Qiantang District, 310020 Hangzhou, Zhejiang Province China; 3 Chuzhou Hospital of Integrated Chinese and Western Medicine, 788 Huifeng East Road, Langya District, 239000 Chuzhou, Anhui Province China

**Keywords:** Direct anterior approach, Position, Fluoroscopy, Acetabular prosthesis, The femoral prosthesis

## Abstract

**Purpose:**

The position of the acetabular and femoral components is critical for stability and wear resistance. The aim of this study is to investigate whether the fluoroscopy-guided direct anterior approach in the supine position (S-DAA) is more helpful in improving the position of acetabular and femoral components than the fluoroscopy-guided direct anterior approach in the lateral decubitus position (L-DAA).

**Methods:**

A retrospective analysis of 76 cases of fluoroscopy-guided direct anterior approach total hip arthroplasty (38 cases in the S-DAA and 38 cases in the L-DAA group) was performed in one hospital from 2019 to 2021. The differences in inclination, anteversion, femoral offset (FO), global offset (GO), and leg length discrepancy (LLD) measurements during and after surgery were analyzed. The postoperative femoral offset (FO), global offset (GO), leg length discrepancy (LLD), and preoperative and postoperative Harris hip score were compared between the two groups.

**Results:**

In the S-DAA group, there were no significant differences in the mean intraoperative inclination angle anteversion angle, FO, GO, and LLD compared to the postoperative values, whereas in the L-DAA group, there were significant differences between the intraoperative and postoperative measurements (P < 0.001, P = 0.009, P＜0.001, P＜0.001 and P = 0.008, respectively). Additionally, there were significant differences in the accuracy of LLD, FO, and GO between the two groups (P < 0.001). Compared with the L-DAA group, the average differences of inclination, anteversion, LLD, FO, and GO during and after operation in the S-DAA group were smaller, and the consistency was higher. There was a significant difference in Harris hip score between the two groups at 1 week after surgery (P = 0.033). There was no significant difference in Harris hip score between 1 month and 3 months after surgery (P = 0.482 and P = 0.797, respectively).

**Conclusions:**

In the supine group, the direct anterior approach (DAA) provides more accurate positioning of the acetabular and femoral components. However, there was no significant difference in hip joint function and activity between the two groups at follow-up.

## Introduction

Total hip arthroplasty (THA) is one of the most successful and reliable orthopedic procedures ever performed [1, 2], and the demand for THA continues to grow given its success and improved quality of life for patients, where the accurate placement of the acetabular component is both important and difficult to achieve [3, 4]. Accurate positioning of the acetabular component in total hip arthroplasty can reduce the occurrence of postoperative complications, such as increasing the stability of the hip joint, slowing the wear of the polyethylene liner, reducing the risk of prosthesis loosening and the difference in bilateral leg length [4, 5]. In addition, Harrison et al. [6] and Lewinnek et al. [7] described the influence of inclination and anteversion Angle on dislocation. Therefore, Lewinnek proposed a “safe zone” in 1978 with an inclination angle of 30°-50° and an anteversion angle of 5°-25°. Beyond this target area, the dislocation rate and biological stress of the cup tends to increase. In addition, the stability and wear resistance tends to decrease [8, 9]. Ideally, these safe zones do not fully reflect the variability and dynamics of true pelvic orientation, including pelvic anatomical plane and pelvic tilt, and are unique to each patient. The final positioning of the acetabular component depends not only on intraoperative positioning but also on functional pelvic tilt and sagittal balance [10].

In addition, global offset(GO), femoral offset(FO), and leg length discrepancy(LLD) are important considerations in THA. Global offset is the sum of femoral and acetabular offsets. Femoral offset is the distance from the center of the femoral head to the femoral shaft in a coronal projection, with an average offset of 41 to 44 mm. Acetabular offset is the perpendicular distance from the center of rotation of the femoral head to the vertical trans-teardrop line. The center of the pelvis is determined by femoral and acetabular offsets [11]. It has been shown that global offset was found to be negatively correlated with abductor function [12, 13]. In addition, leg length discrepancy is also common after total hip arthroplasty, which often causes patient dissatisfaction, and can lead to sciatica, gait disturbance, and the possibility of aseptic loosening in the later stage. The average discrepancy ranges from 3 to 17 mm, and most of the differences are within 10 mm [11].

Experienced surgeons determine the placement of the prosthesis during THA by using traditional methods that make use of mechanical guides and bony anatomical landmarks such as the anterior superior iliac spine, the pubic symphysis, and/or the transverse acetabular ligament [14]. Molho et al. [15] showed that the accuracy of acetabular component position could be improved only by identifying the anatomical position of the transverse acetabular ligament. Although the surgeon is familiar with and can recognize the anatomical landmarks of the bone, it is still difficult to grasp the accuracy of the position of the acetabular and femoral components during the operation. Because this is also related to the intraoperative position of the patient and the position of the operator on the operating Table [16].

With the development of the medical industry, there are more and more methods to improve the positioning of prostheses, such as fluoroscopy [8, 17, 18], computer navigation [19, 20], and robotic assistance [21, 22] to help surgeons better determine the position of prostheses. Among them fluoroscopy is the most practical, economical and widely used method [9]. In the direct forward approach to total hip replacement, the procedure is performed in the supine position, making it easier to use C-arm fluoroscopy during the operation to evaluate and adjust the position of the femur and acetabular prosthesis. In a study comparing the anterior and posterior approaches, Slotkin et al. [10] demonstrated that the use of fluoroscopy in the direct anterior approach reduced the position variability of the acetabular prosthesis in supine patients compared to the posterior unguided approach. Beamer et al. [23] found that the odds of placing the cup in the Lewinnek safe zone for inclination angle and anteversion angle were 2.3 times greater with the use of fluoroscopy, compared to the surgeon’s experience of placing an acetabular cup by hand, compared to the surgeon’s experience of placing an acetabular cup by hand. Belyea et al. [24] found that the use of fluoroscopy in the posterior approach increased the accuracy of cup inclination angle and femoral offset compared with the unguided posterior approach. In the study of robot-assisted or computer-guided THA, Zhou et al. [4] compared robot-assisted THAs with conventional THAs without robotic technology and found that robot-assisted technology can assist surgeons with implanting more acetabular cups into the safe zone. Nishihara et al. [19] found that the use of a CT-based navigation system can improve the position of the acetabular cup compared to the free-hand placement of the cup.

In recent years, several minimally invasive methods have been generated, among which the direct anterior approach (DAA) for total hip arthroplasty is favored by physicians [25, 26]. DAA is usually performed on patients in the supine position (S-DAA), and although DAA can also be performed on patients in the lateral position (L-DAA), the study of the difference between these two positions is insufficient. To the best of our knowledge, previous studies compared the influence of intraoperative fluoroscopy on the position of the total hip prosthesis through the direct anterior approach and the posterior approach, and the influence of non-fluoroscopic guidance and intraoperative fluoroscopy on the position of the total hip prosthesis [9, 23, 24]. There is only few literature comparing whether the patient position has an effect on the position of the prosthesis under fluoroscopic guidance under the same surgical approach. This study aimed at comparing the effect of different patient positions on the accuracy of the implant position during the direct anterior approach. We hypothesized that the DAA in the supine position under fluoroscopy would be more accurate in positioning the implant than the DAA in the lateral position under fluoroscopy.

## Materials and methods

This study was approved by our institutional review board(approval number:2022-K-337-01), and informed consent was obtained from all patients. For this retrospective comparative study, data on THA performed by a senior surgeon at a single center between 2019 and 2021 were reviewed. Inclusion criteria restricted the study to patients with an age of 18 years or older who underwent unilateral primary total hip arthroplasty for avascular necrosis of the femoral head, femoral neck fracture, osteoarthritis, or secondary arthritis. patients were excluded if the index procedure was bilateral THA, the contralateral hip had previously been replaced, history of prior trauma or surgery to the hips and developmental dysplasia of the hip. All THAs in this study were performed using DAAs with an uncemented hemispherical acetabular component (Triden®PSL, X3™ Polyethylene liners; Stryker, Kalamazoo, Michigan) and an uncemented tapered wedge femoral component(Accolade TMZF® with Biolox® Delta ceramic head;Stryker, Kalamazoo, Michigan).

**Fig. 1:**
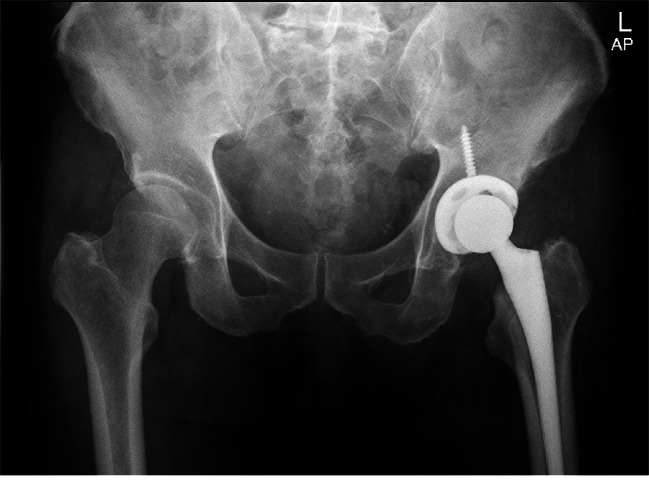
A standard postoperative AP pelvis X-ray

**Fig. 2:**
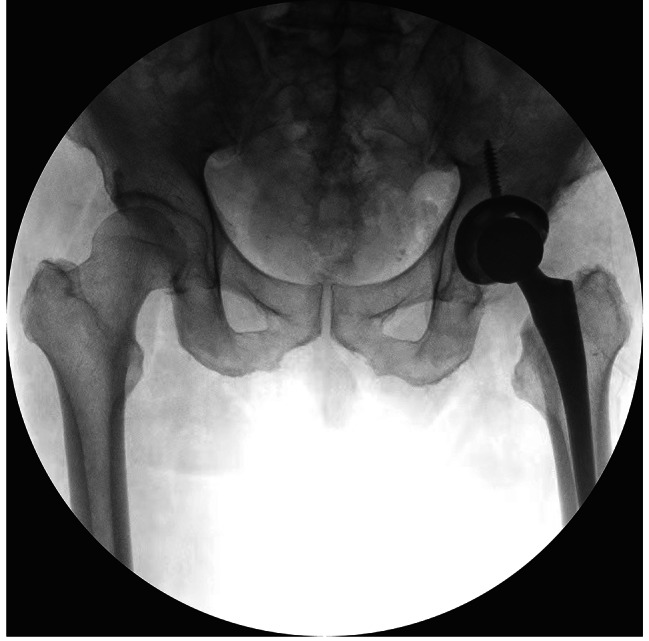
A fluoroscopic image was obtained with the C-arm machine, while the shape and position of the obturator foramen and the pubic symphysis was observed by the surgeon

**Fig. 3:**
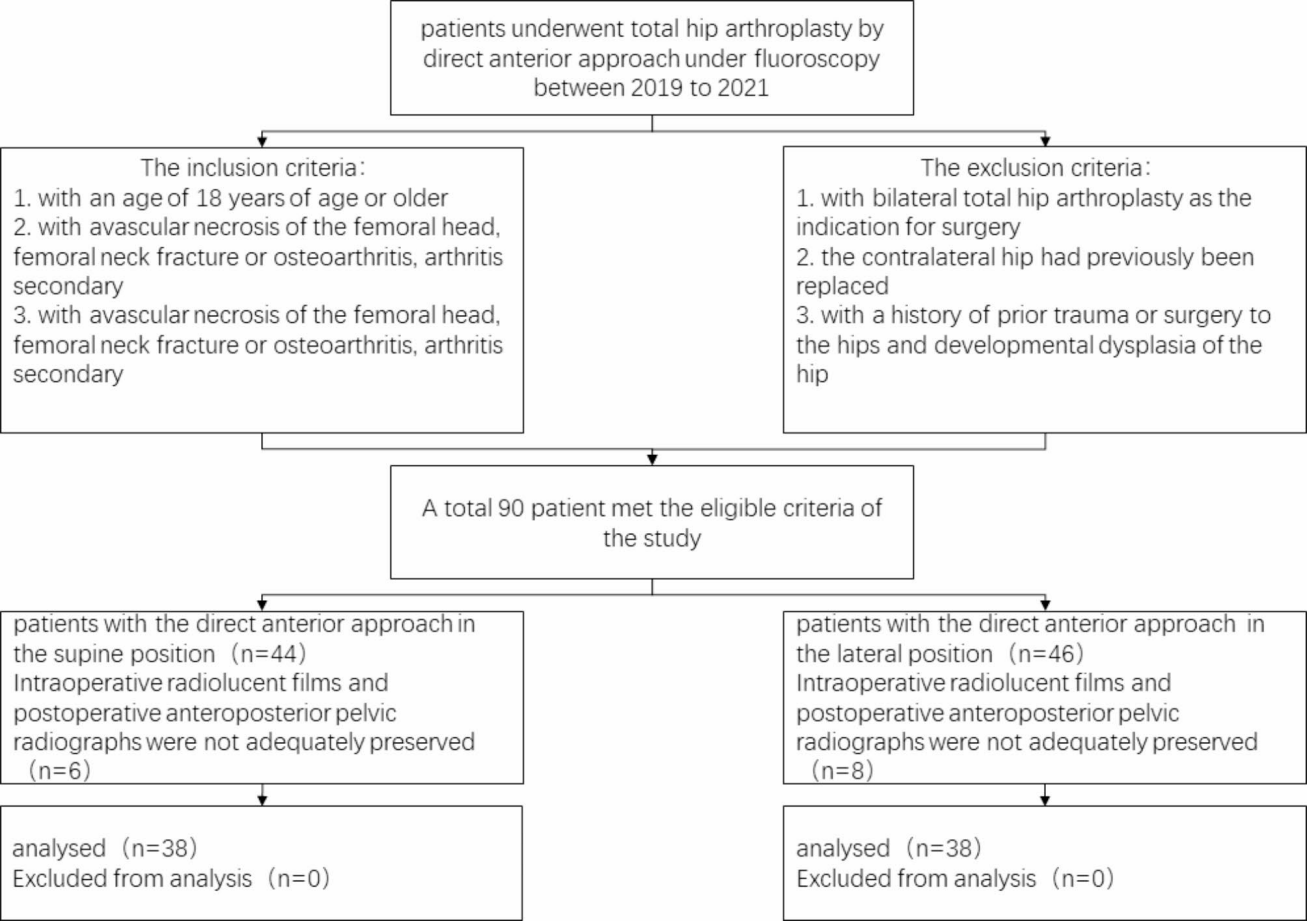
Study flow chart

Information was obtained from the electronic medical record(EMR) for each patient, including the laterality of the surgically treated hip, the surgeon performing the procedure, the patient’s age, sex, body mass index (BMI), surgical procedure, and preoperative diagnosis, among others. Upright digital anteroposterior pelvic radiographs were obtained 6 weeks after surgery (Fig. 1) and Intraoperative fluoroscopy films (Fig. 2). The intraoperative fluoroscopy films were obtained by the C-arm machine, and one intraoperative fluoroscopy film deemed clear by the surgeon was retained. The hips without a clear intraoperative fluoroscopy film and a clear standard postoperative anterior-posterior (AP) pelvis X-ray were excluded. We reviewed a consecutive number of patients with S-DAA. Out of the first 44, 38 had a clear intraoperative fluoroscopy film and a clear standard postoperative AP pelvis X-ray and were included. Working back in time, We also consecutively reviewed 46 patients with L-DAA to find 38 with a clear intraoperative fluoroscopy film and a clear postoperative AP pelvis X-ray. This left a total of 76 patients which were available for inclusion in this study (Fig. 3).

**Fig. 4:**
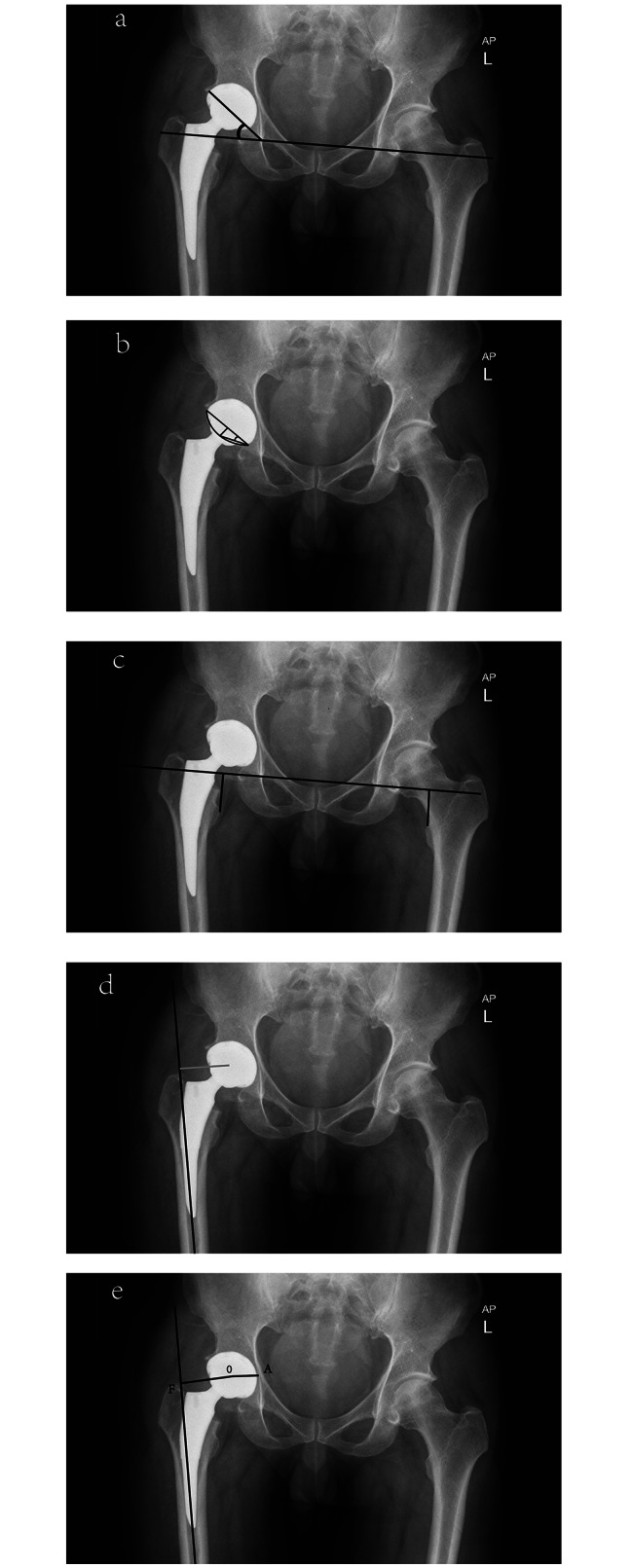
**(a)** Acetabular cup inclination; **(b)** Acetabular cup anteversion; **(c)** leg length discrepancy (LLD); **(d)** femoral offset (FO); **(e)** global offset (GO)

The S-DAA was performed with the patient in the supine position on a radiolucent table, which facilitates intraoperative manipulation using a C-arm fluoroscope above and below the patient to aid in implant positioning. The L-DAA was performed with the patient in the decubitus position. For the L-DAA, the patient uses hammered sacral and pubic symphysis positioning system, and these pelvic locators were angled so that they did not interfere with the large C-arm AP pelvic fluoroscopy images as they pass through the operating table. Intraoperative fluoroscopic guidance was performed according to the procedure described by Ji and Stewart [9], and the placement of acetabular and femoral components was indicated by fluoroscopy. The acetabular inclination and anteversion angles, LLD, FO, and GO were measured (Fig. 4). The neutral position of the hip joint was taken before and after surgery, and the x-ray beam was centered on the pubic symphysis. In addition, preoperative and postoperative Harris hip scores were recorded and evaluated in both groups.

### Radiographic measurements

Radiographic evaluation was performed on anteroposterior radiographs of the hip at 6 weeks after surgery and Intraoperative fluoroscopy films. Measurements were made using picture archiving and communication system (PACS) software (version 6.0, Fujifilm, Stanford, CT, USA).

1. Acetabular inclination angle (Fig. 4a). The angle between the upper and lower margins of the acetabulum and the bilateral teardrops was measured on a standard weight-bearing AP slice.
2. Acetabular anteversion angle (Fig. 4b). The angle formed between the acetabular axis and the coronal plane was measured by viewing on a standard weight-bearing AP radiograph [27].
3. Leg length discrepancy (Fig. 4c). The difference in the vertical distances between the line connecting each pelvic tear to each lesser trochanter of the femur was observed on a standard weight-bearing AP slice and compared to the contralateral side [13].
4. Femoral offset (Fig. 4d). Observed on a standard weight-bearing AP film, measuring the distance between the longitudinal anatomical axis of the femur and the center of rotation of the femoral head, as compared to the contralateral side. [11, 12, 24, 28]. Measurement was done on both sides, and the FO of the operated side was compared with that of the unoperated side.
5. Global offset (Fig. 4e). Observation on a standard weight-bearing AP film, the femoral offset was added to the distance from the center of the femoral head to a perpendicular line passing through the medial border of the ipsilateral teardrop point of the pelvis (AO) [29–31]. Measurement was done on both sides, and the GO of the operated side was compared with that of the unoperated side.

### Statistical analysis

A Kolmogorov–Smirnov test was used to evaluate for normality of data. Student’s t test was used to evaluate normally distributed continuous variables and a Mann–Whitney U test to compare other types of continuous data between groups. Categorical variables were compared using the Chi-Squared test between group differences. All statistical analyses were performed using SPSS Statistical Software (version 27.0, SPSS Inc., Armonk, New York). Statistical significance was set at p < 0.05.

## Results

Demographic and perioperative data are shown in Table 1. There were no significant differences in age, gender, surgical side, or body mass index between the two groups (S-DAA vs. L-DAA). Table 2 summarizes the mean acetabular inclination angle values and anteversion angle values for the two groups. Student’s t-test showed that there was a statistically significant difference between the intraoperative and postoperative measurements of inclination angle and anteversion angle in the L-DAA group (P＜0.001 and P=0.009, respectively), whereas there was no statistically significant difference in the S-DAA group. For the differences in FO, GO, and LLD between the two groups, the results are summarized in Table 3, and statistically significant differences between L-DAA and S-DAA were observed (P < 0.001). The Harris hip score of the two groups before surgery, 1 week after surgery, 1 month after surgery, and 3 months after surgery were compared, and the results are shown in Table 4. Student’s t-test showed that there was a significant difference in the Harris hip score between the two groups 1 week after surgery (P = 0.033), while there was no significant difference in Harris hip score 1 month and 3 months after surgery (P = 0.482 and P = 0.797, respectively).Mean FO, GO and LLD values within the two groups are summarised in Table 5. The Student’s test showed that the FO and GO in the L-DAA group were significantly different during and after the operation (P < 0.001 and P = 0.008). Additionally, the Mann-Whitney U test showed that the Leg length discrepancy in the lateral position group was significantly different during and after operation (P < 0.001), in this group. No statistically significant differences wer observed for these measurements, in the S-DAA group.

**Table 1:** Demographic features of the study group

Variables	S-DAA	L-DAA	*P* value
Mean age	62.3 ± 11.6	63.5 ± 9.9	0.641^a^
Gender			
Male Female	17 21	19 19	0.646^b^
Side			
Right Left	20 18	21 17	0.491^b^
Mean BMI	22.9 ± 2.1	23.1 ± 1.6	0.789^a^

S-DAA: direct anterior approach in the supine position, L-DAA: direct anterior approach in the lateral position, BMI: body mass index

^a^ Student’s t test

^b^ Chi-squared test

**Table 2:** Mean, standard deviation, and variances for cup inclination and anteversion

Variables	Intraoperative fluoroscopy	Postoperative standing AP X-ray	*P* value
S-DAA			
Inclination Anteversion	40.6 ± 2.89 16.2 ± 1.88	40.9 ± 2.84 16.4 ± 2.43	0.265^a^ 0.637^a^
L-DAA			
Inclination Anteversion	37.6 ± 2.33 17.5 ± 3.34	40.3 ± 2.74 16.1 ± 2.97	＜0.001^a^ 0.009^a^

S-DAA direct anterior approach in the supine position, L-DAA direct anterior approach in the lateral position

^a^ Student’s t test

**Table 3:** The mean and standard deviation of femoral offset, global offset, and absolute difference of Leg length discrepancy after postoperative in S-DAA and L-DAA were described

Variables	S-DAA	L-DAA	*P* value
Femoral offset difference(mm)	2.4 ± 0.69	3.2 ± 0.47	＜0.001^a^
Global offset difference(mm)	1.1 ± 0.46	1.6 ± 0.51	＜0.001^a^
Leg length discrepancy(mm)	2.1 ± 0.42	4.3 ± 0.43	＜0.001^b^

S-DAA direct anterior approach in the supine position, L-DAA direct anterior approach in the lateral position

^a^ Student’s t test

^b^ Mann–Whitney U test

**Table 4:** Harris Hip Score before surgery, 1 week after surgery, 1 month after surgery, and 3 months after surgery in both groups

Variables	S-DAA	L-DAA	*P* value
Preoperative	39.3 ± 3.74	39.1 ± 3.56	0.827^a^
Postoperative			
One week One month Three month	68.7 ± 3.00 77.7 ± 2.71 87.3 ± 3.01	67.1 ± 3.63 77.3 ± 2.47 87.1 ± 3.21	0.033^a^ 0.482^a^ 0.797^a^

S-DAA direct anterior approach in the supine position, L-DAA direct anterior approach in the lateral position

^a^ Student’s t test

**Table 5:** Mean, standard deviation for femoral offset difference, global offset difference, and Leg length discrepancy

Variables	Intraoperative fluoroscopy	Postoperative standing AP X-ray	*P* value
S-DAA			
Femoral offset difference(mm) Global offset difference(mm) Leg length discrepancy(mm)	2.6 ± 0.60 1.2 ± 0.50 2.2 ± 0.45	2.4 ± 0.69 1.1 ± 0.46 2.1 ± 0.42	0.399^a^ 0.433^a^ 0.509^b^
L-DAA			
Femoral offset difference(mm) Global offset difference(mm) Leg length discrepancy(mm)	2.7 ± 0.48 1.3 ± 0.40 4.8 ± 0.46	3.1 ± 0.47 1.56 ± 0.51 4.3 ± 0.43	＜0.001^a^ 0.008^a^ ＜0.001^b^

S-DAA direct anterior approach in the supine position, L-DAA direct anterior approach in the lateral position

^a^ Student’s t test

^b^ Mann–Whitney U test

**Fig. 5:**
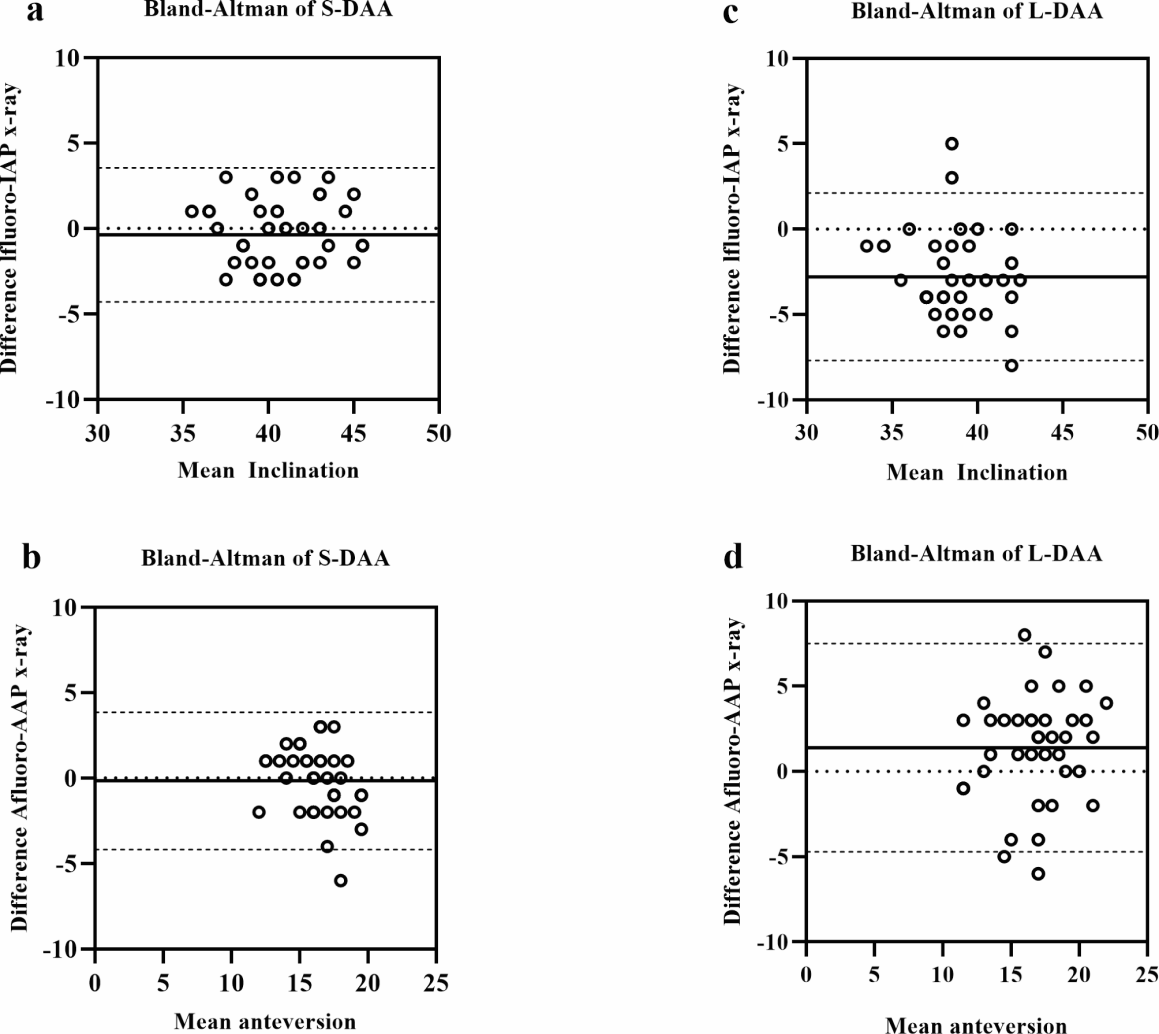
Deviation of the intraoperative fluoroscopic values from postoperative standing AP pelvis values compared to their mean. **(a)** Direct anterior approach in supine position (S-DAA) inclination. **(b)** S-DAA anteversion. **(c)** The direct anterior approach in lateral position (L-DAA) inclination. **(d)** L-DAA anteversion. The dotted lines represent the 95% limits of agreement (Bland-Altman graph)

**Fig. 6:**
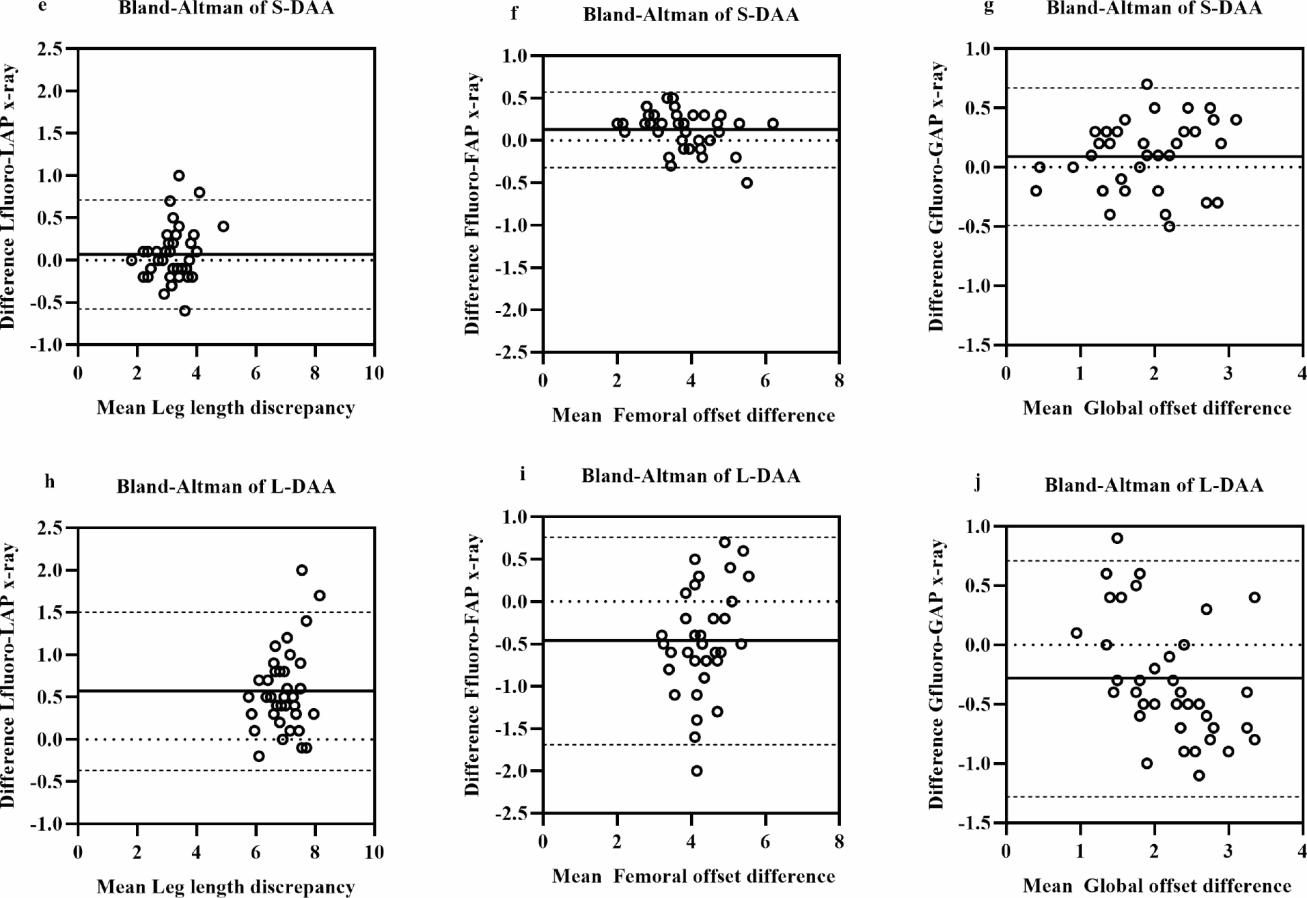
Deviation of the intraoperative fluoroscopic values from postoperative standing AP pelvis values compared to their mean. **e**. Direct anterior approach in supine position (S-DAA) leg length discrepancy. **f**. S-DAA femoral offset difference. **g**. S-DAA global offset difference. **h**. The direct anterior approach in lateral position (L-DAA) leg length discrepancy. **i**. L-DAA femoral offset difference. **j**. L-DAA global offset difference. The dotted lines represent the 95% limits of agreement (Bland-Altman graph)

In addition, the accuracy and bias of intraoperative fluoroscopy were evaluated for both groups and a case-by-case comparison was performed, with the mean differences and 95% confidence intervals for each of these measures shown in Figs. 5a-d and 6e-j in terms of Bland-Altman diagrams. In the supine group, the mean intra- and post-operative difference in inclination was − 0.4 ± 2.01° (95% [CI]-4.3 to 3.6°); the mean intra- and post-operative difference in anteversion was − 0.2 ± 2.05° (95% [CI]-4.2 to 3.9°); the mean intra- and post-operative difference in LLD was 0.1 ± 0.33 mm (95% [CI]-0.6 to 0.7 mm); the mean intra- and post-operative difference in FO was 0.1 ± 0.23 mm (95% [CI]-0.3 to 0.6 mm); and the mean intra- and post-operative difference in GO was 0.1 ± 0.30 mm (95% [CI]-0.5 to 0.7 mm). In the lateral group, the mean intra- and post-operative difference in inclination was − 2.8 ± 2.51° (95% [CI]-7.7 to 2.1°); the mean intra- and post-operative difference in anteversion was 1.4 ± 3.12° (95% [CI]-4.7 to 7.5°); the mean intra- and post-operative difference in LLD was 0.6 ± 0.48 mm (95% [CI]-0.4 to 1.5 mm); the mean intra- and post-operative difference in FO was − 0.46 ± 0.62 mm (95% [CI]-1.69 to 0.76 mm); and the mean intra- and post-operative difference in GO was − 0.28 ± 0.51 mm (95% [CI]-1.28 to 0.71 mm).

## Discussion

Total hip arthroplasty (THA) is one of the most effective orthopedic procedures for the treatment of end-stage hip osteoarthritis, providing patients with sustained pain relief and functional recovery. An important goal for the success of total hip arthroplasty is to accurately reconstruct the biomechanics and geometry of the hip joint, including the range of motion and functional outcome. Among these, the position of the acetabular and femoral components is very important for prosthesis loosening, wear, hip joint loading, abductor strength, range of motion, reduction of dislocation risk and wear resistance [32–34].

In recent years, DAA has become increasingly popular because it causes less muscle damage, has a faster recovery, and is less prone to hip dislocation [35, 36]. DAA includes both L-DAA and S-DAA, and few studies have used DAA to directly compare total hip arthroplasty in these two different positions. Although the supine position is the preferred position for DAA by most surgeons, some surgeons still choose the lateral position. With the development of science and technology, a variety of techniques have been introduced to improve the accuracy of prosthesis positioning, of which intraoperative fluoroscopy is the most widely used. More and more evidence has shown that intraoperative fluoroscopy can improve the position of the cup implant, thus preventing prosthesis dislocation [35, 37]. In this study, there were statistically significant differences between the intraoperative inclination and anteversion of fluoroscopy and postoperative inclination and anteversion of fluoroscopy in the L-DAA group, whereas there was no significant difference in the S-DAA group. Because the patient’s surgical position will directly affect the orientation of the pelvis, the change in the orientation of the pelvis may lead to the dislocation of the acetabular prosthesis, which affects the measurement of acetabular position after operation [10, 38]. In addition, recognition of preoperative pelvic tilt is necessary to accurately determine component position intraoperatively [39]. Therefore, when the patient is in the supine position, the pelvic orientation changes less during the operation. The use of fluoroscopy helps to simulate the pelvic tilt before the operation, and provides feedback on the pelvic orientation and the position of the cup, which improves the accuracy of the surgeon in placing the acetabular component in the safe zone [36]. However, Maeda et al. also pointed out that there was no significant difference in cup angle accuracy between DAA in the lateral decubitus position and DAA in the supine group after the use of a mechanical guide [40].

One of the important goals of primary total hip arthroplasty is to restore limb length parity. Leg length discrepancy can lead to residual buttock pain, low back pain, and other patient discomforts. [11, 41] This study compared the absolute difference in leg length between the two groups after surgery, and the difference was statistically significant. The intra-group comparison found that the average difference of intraoperative and postoperative LLD in S-DAA was smaller than that in L-DAA, and its consistency was higher. The length of the lower limb by fluoroscopy should be measured according to the height of the lesser trochanter and ischial tuberosity or the height of the greater trochanter and acetabular rotation center of the hip joint. In S-DAA, the pelvic position is relatively fixed and not easy to change, which makes the difference between intraoperative and postoperative acetabular position changes small. The supine position is convenient for C-arm fluoroscopy to obtain images so that the surgeon can visually compare the leg length of both sides so that the component positions and measurement results are more accurate. In L-DAA, the pelvic direction changes more, which makes the image obtained by intraoperative and postoperative fluoroscopy quite different [42, 43]. Thus, L-DAA has a lower accuracy in the position of the prosthesis, resulting in a significant difference in the absolute postoperative limb length difference. Renkawitz et al. [29]. also found that the leg length discrepancy after THA should be avoided to be ≥ 5 mm, and the preoperative leg length should be maintained or restored to the contralateral leg length, which is conducive to the stability of the prosthesis. Global offset is also an important step after total hip arthroplasty. Worlicek et al. [44] found that the global offset was related to the incidence of femoral trochanteric pain in patients. When the global offset was greater than 5 mm, the incidence of femoral trochanteric pain was higher. It has also been found that excessive femoral offset has adverse effects on patients’ postoperative gait and affects their quality of life [29, 45, 46]. We found that the global offset difference in the supine group was smaller than that in the lateral group and intra-group intraoperative and postoperative comparisons contained less bias in the supine group. This indicates that the direct anterior approach in the supine position is less likely to cause an increase in the offset, which allows more accurate in positioning the prosthesis under fluoroscopy and reduces the risk of postoperative pain for patients. This is conducive to postoperative functional rehabilitation exercises. We also compared the Harris hip score between the two groups. It was slightly higher in the supine group than in the lateral decubitus group 1 week after surgery. In addition, there was no significant difference in the Harris Hip Score between the two groups at 1 month and 3 months postoperatively. The patients had significant improvement in hip range of motion and function, and their quality of life was also improved.

Although our study showed that DAA in the supine position under fluoroscopy was more accurate for prosthesis positioning, there are still some shortcomings in this study. First, the sample size in this study is too small, and the results may be biased, so further studies with large sample sizes are still needed. Second, the BMI of the patients included in the study was not classified as ultra-obese, and the position of ultra-obese patients was not investigated, because it has been suggested that [43] for S-DAA, obese or strong patients have accumulated a large amount of muscle or adipose tissue around the incision, which makes the surgeon’s surgical vision blurred, while for L-DAA, due to gravity, muscle or adipose tissue will move away from the incision, so that the surgical field can be fully exposed, so relevant clinical studies on obese patients are still needed. Third, the accuracy of anteversion measurements with X-rays is low. No comparison with navigation methods (or other techniques) was made. Fourth, the selection of intraoperative and postoperative film quality is subjective. In addition, this study is only a single-center study, and we will further refine these shortcomings in future studies.

## Conclusion

The DAA in the supine position is more accurate for positioning the prosthesis than the DAA in the lateral position under fluoroscopy. In the follow-up, there is no significant difference in the range of motion and function of the hip joint, and the quality of life is improved in both patients in the supine position and patients in the lateral position after total hip arthroplasty with DAA.

## Data Availability

The datasets used and analyzed during the current study are available from the corresponding author upon reasonable request.

## Code Availability

The article does not cover any code.
